# Leveraging microfluidic confinement to boost assay sensitivity and selectivity†

**DOI:** 10.1039/d5sc00199d

**Published:** 2025-03-11

**Authors:** Shaoyu Kang, Jason J. Davis

**Affiliations:** a Department of Chemistry, University of Oxford South Parks Road Oxford OX1 3QZ UK jason.davis@chem.ox.ac.uk +44(0) 1865272690 +44(0) 1865275914

## Abstract

The native and tunable microscale fluid manipulation accessible within 3D-printed configurations can be a transformative tool in biosensing, promoting mass transport and sample mixing to boost assay performance. In this study, we demonstrate that channel height restrictions can support a 2000% acceleration in target recruitment kinetics, a notable 600% improvement in target response magnitude, and a 300% enhancement in assay selectivity within an entirely reagentless format that requires neither catalytic amplification nor the employment of specialized nanomaterials. This highly accessible experimental configuration supports robust target detection from serum at simple, untreated, and un-passivated sensor surfaces. The underlying operational principles have been elucidated through a combination of theoretical analysis and COMSOL simulation; the enhanced analyte flux leveraged by channel confinement is directly responsible for these effects, which also scale with both bioreceptor surface density and target binding affinity. The operational simplicity of this assay format with its resolved channel and flux promoted assay performance, holds significant value not only for biosensing but also for broader microfluidic-integrated applications, such as biosynthesis and catalysis.

## Abstract 2

The native and tunable microscale fluid manipulation accessible within 3D-printed configurations can be a transformative tool in biosensing, promoting mass transport and sample mixing to boost assay performance. Response speed, magnitude and selectivity.

## Graphical abstract

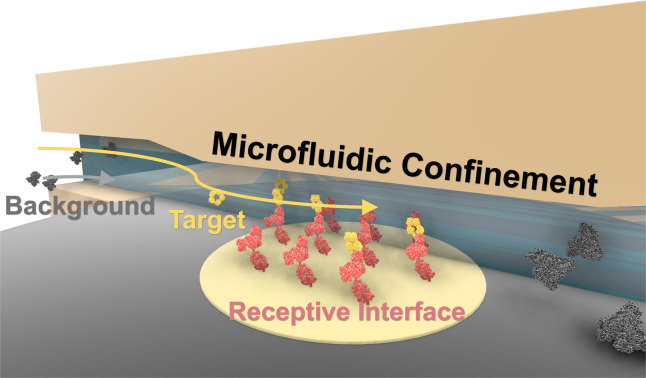

## Introduction

Biomarkers are molecular indicators that reflect a wide range of physiological disease states.^1,2^ Accurate quantification of these holds a pivotal and growing role in clinical practice,^3^ enabling early disease detection, much more effective intervention, and continuous monitoring throughout treatment (thereby optimizing therapeutic strategies).^4^ Interfacial biosensors facilitate this by integrating surface-bound bioreceptors within transducing configurations that effectively convert biorecognition events into quantifiable signals.^5,6^ Those which are electrochemical are particularly favored for their rapid response times, simplicity of operation, cost-effectiveness, and compatibility with on-site device integration.^7–9^ Indeed, since the development of the electroanalytical blood glucose biosensor,^10,11^ these platforms have supported applications spanning clinical pathogen detection,^9,12–15^ wearable sensors,^16–19^ and environmental monitoring.^20–24^

Marker detection is, by default, associated with its recruitment at a planar (electrode) surface. This, however, comes with limitations associated with the kinetics and scale of specific signal development relative to background (nonspecific fouling).^25,26^ The sluggish mass transport of targets to these interfaces exacerbates this issue,^27^ as it blurs the distinction between specific and nonspecific binding. A range of amplification strategies,^28–30^ typically involving either enhancement of the electrochemical surface area through the incorporation of nanomaterials (*e.g.*, nanoparticles, graphene),^31–35^ or the employment of catalytic and chain reactions to magnify the signals, have accordingly become common, along with the utilization of “double recognition” sandwich assays as a means of improving specificity.^36–40^ While these approaches can be effective, the necessary integration of nanomaterials, catalysts, redox tags, enzyme-modified secondary antibodies *etc.*, increases assay cost, complexity, and, significantly, can undermine reproducibility through the number of variables associated with the multi-step protocols and repeated washing steps therein.

Microfluidic configurations have increasingly been employed in biosensing applications,^41–46^ in part to reduce sample volumes, streamline workflow, and minimize experimental variability.^47^ In previous work, building on a well-established bio-receptive polyaniline (PANI) interface,^48,49^ we have introduced an on-chip integrated capacitive biosensor capable of assessing binding kinetics in real-time in a reagentless and label-free manner. By monitoring the temporal changes in the PANI capacitive fingerprint, *C*_r_, we have further demonstrated that an analysis of the kinetics of target recruitment can itself support marker quantification in just 15 seconds, with a picomolar Limit of Detection (LoD). In these microfluidic configurations, sample delivery is predominantly governed by a strong convective component,^50,51^ as illustrated in eqn (1).

1 *J*_channel_ = *J*_diff_ + *J*_conv_ = −*D*∇*c*_A,b_ + *c*_A,b_·*U*

where the channel flux *J*_channel_ (mol cm^−2^ s^−1^) is defined as the number of moles passing through a unit area per unit time, with diffusional *J*_diff_ and convective *J*_conv_ components. *D* is the diffusion coefficient of the analyte, expressed in cm^2^ s^−1^; *c*_A,b_ is the concentration of analyte in the bulk solution; ∇ denotes the analyte concentration gradient; *U* is the flow velocity in cm s^−1^. This strong convection enhances mass transport,^51^ and, under laminar flow conditions (Reynolds number, Re < 2300),^52^ the associated flux is also directly dependent on analyte bulk concentration, with a proportionality constant *k*_Lev_, as shown in eqn (2).

2 *J*_channel_ = *k*_Lev_·*c*_A,b_

The mass transport coefficient *k*_Lev_, is highly sensitive to fluid properties (*e.g.*, viscosity, flow rate) and channel geometry, as described by the Levich equation (eqn (3)).^50^ Here, *V*_f_ represents the volume flow rate (cm^3^ s^−1^), *A* the reactive area, and *h* the half-height of the channel in cm.3
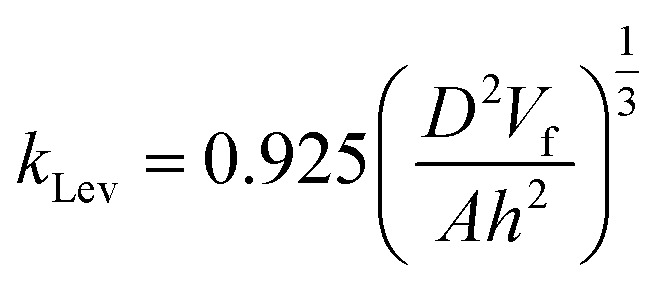


**Fig. 1:**
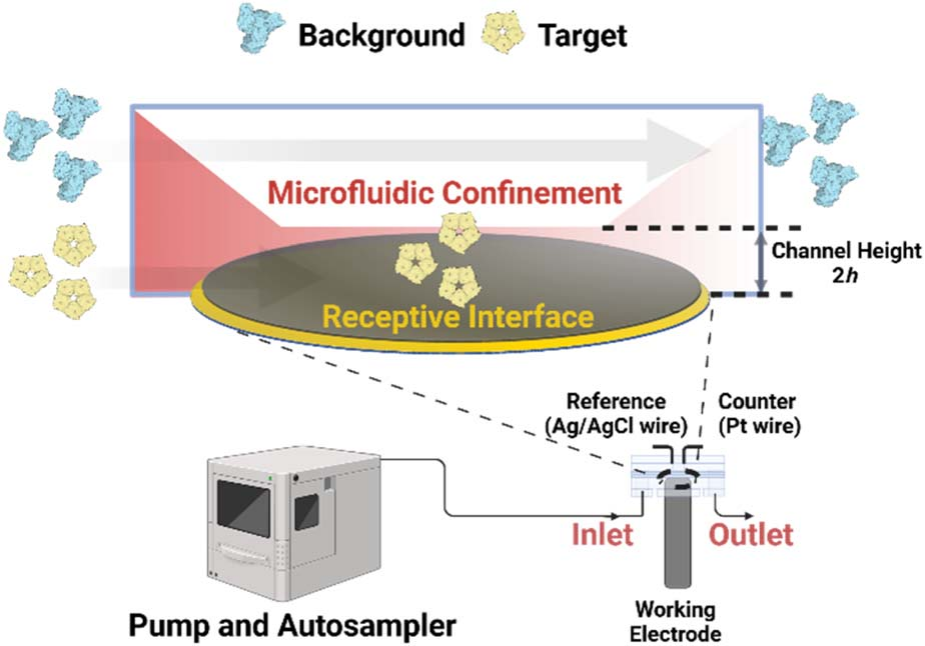
Schematic representation of utilizing microfluidic confinement to boost assay sensitivity and the selectivity of target recruitment. The working disc electrode is inserted into a custom 3D-printed microfluidic cell with confined channel height, accessible ranging from 1000 μm to 20 μm. Samples are delivered at a controlled flow rate by an automated syringe pump. A red-color gradient within the channels illustrates analyte flux toward the bio-receptive interface, with darker shades indicating higher flux. Within more confined volumes, increased convective flow enhances mass transport, leading to the rapid accumulation of analytes on the bio-receptive surface and a significantly increase in assay sensitivity. This process, governed by association rate constants, significantly improves selectivity of targeted recruitment by concentrating analytes at the detection interface while minimizing nonspecific background interference.

Since interfacial binding of a target is a series/sequential combination of transport and specific binding, and the latter is typically associated with a rate constant *k*_on_ ≥ 10^5^ M^−1^ s^−1^,^53,54^ the target accumulation rate is inherently flux-limited. The magnitude of mass transport coefficient *k*_Lev_ can, therefore, profoundly influence the overall kinetics of analyte accumulation on a reactive surface ∂*c*_A–B,s_/∂*t*,^55,56^ as expressed in eqn (4). Here, *k*_on_ is the specific binding association rate constant, and *c*_B,s_ represents the receptor surface coverage.4
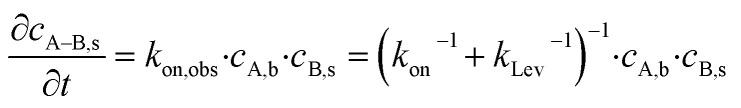
Herein, we have attempted to engineer 3D-printed microfluidic confinement with channel heights reduced to just a few micrometers in order to enhance convective flux and thus *J*_channel_. It was anticipated that this would, in turn, facilitate more rapid target accumulation at the bio-receptive interface.^57–59^ The resulting acceleration in binding kinetics not only enables faster signal development, but also amplifies the equilibrium response and, thus, assay sensitivity. In contrast to high affinity specific target binding, nonspecific surface association is of a much lower surface affinity (slower *k*_on_) and is less affected by changes in flux (as mass transport is less likely to be the rate-determining step). One would accordingly expect flux enhancements to boost the accumulation of specific targets over nonspecific ones (increasing assay specificity). By leveraging microfluidic confinement (Fig. 1), then, we can now demonstrate substantial increases in both assay sensitivity and target selectivity with real-world relevant samples.

## Results and discussion

### Theoretical analysis

Prior to examining the electrochemical data acquired within confined channels, we first established a theoretical framework to underpin the relationship between microfluidic flow dynamics and accumulation at a receptive surface. Within microfluidic channels, the analyte (A) is delivered to the receptor interface through a combination of diffusion and forced convection. Upon reaching the surface, it subsequently binds to the receptor (B) to form a complex A–B, a process describable as:
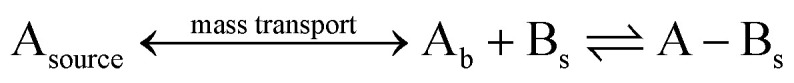
Here, the notations “s” and “b” refer to species at the sensor surface and in the bulk solution, respectively.

The specific signal development *S*_A_ is directly proportional to the surface concentration of the complex A–B, with a proportionality constant *K*_A_,

5 *S*_A_ = *K*_A_·*c*_A–B,s_

Under convective conditions, the observed association rate constant *k*_on,obs_ depends on the intrinsic association rate constant *k*_on_ and the mass transport coefficient *k*_Lev_, expressed as the reciprocal sum.^55,56^

6 *k*_on,obs_ = (*k*_on_^−1^ + *k*_Lev_^−1^)^−1^

The observed equilibrium binding constant *K*_a,obs_ is derived as,7
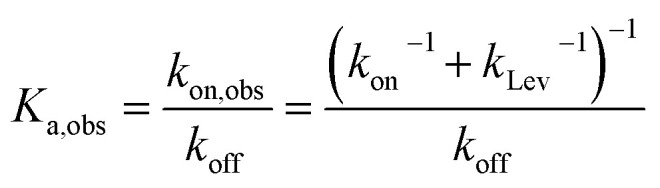


Reducing the channel height increases the mass transport coefficient *k*_Lev_ (eqn (3)), resulting in enhancements in *k*_on,obs_ and, consequently, *K*_a,obs_. Assuming Langmuir adsorption behavior,^60^eqn (5) can be expressed as follows, where *θ* is the fractional coverage of receptors by the analyte.8
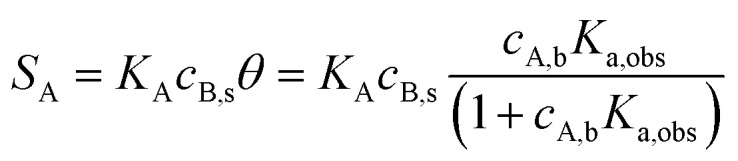


By combining eqn (4) and (5), the rate of signal change over time ∂*S*_A_/∂*t* becomes,9
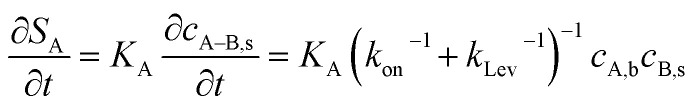


It is evident that both equilibrium *S*_A_ and temporal ∂*S*_A_/∂*t* responses increase with the mass transport coefficient *k*_Lev_, as indicated by positive derivatives,10
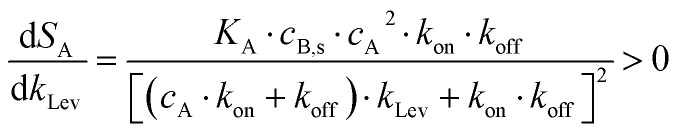
11
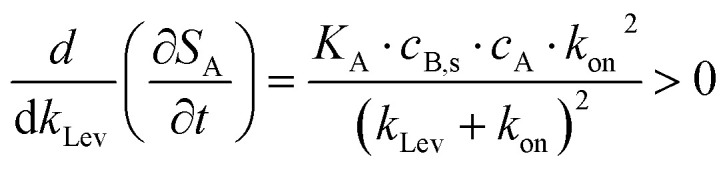


These positive derivatives indicate that increasing *k*_Lev_ by compressing the delivery channel can boost both the magnitude of the signal response and the rate of signal generation.

Selectivity is the ability of a sensor to distinguish the target analyte (A) from background interferent (I),^61^ and can be defined here as the ratio of their rates of signal generation.12
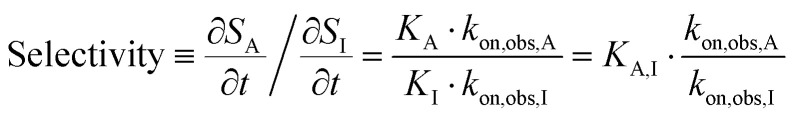


As shown in eqn (12), selectivity depends not only on the selectivity coefficient *K*_A,I_; defined as the ratio of the proportionality constants for the signal development of the specific analyte A to the background I, reflecting the receptor's preference for the target) but also on the association rate constants, which can be modulated by flow dynamics. As discussed earlier, the overall binding kinetics of specific biorecognition events (*k*_on_ ≥ 10^5^ M^−1^ s^−1^) are typically dominated and limited by mass transport (*k*_on,A_ ≫ *k*_Lev_, *k*_on,obs,A_ ≈ *k*_Lev_). While the contribution of enhancements in binding-limited reaction (*i.e.*, nonspecific binding) is negligible—that is, *k*_on,I_ ≪ *k*_Lev_, *k*_on,obs,I_ ≈ *k*_on,I_. Thus, eqn (12) simplifies to,13
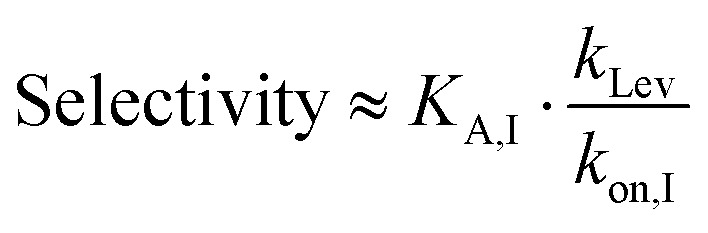


Eqn (13) reveals the origin of enhanced selectivity attainable under microfluidic confinement. By finely controlling flow (*e.g.*, channel height), it is, specifically, possible to optimize the mass transport coefficient *k*_Lev_, and thereafter amplify specific nonspecific surface association. While microfluidic enhancements certainly are expected to accelerate the recruitment of molecules in general by increasing flux, the effect is likely to be comparatively less pronounced for low molecular weight/higher diffusivity molecules, where transport constraints play a less dominant role.

### COMSOL simulations

**Fig. 2:**
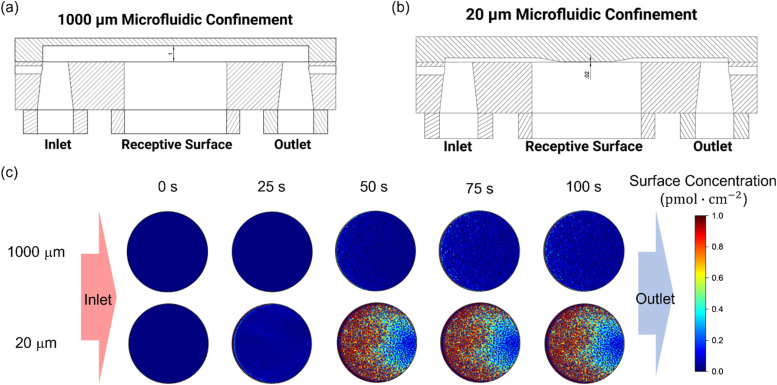
Schematic representation of the COMSOL simulation model for (a) 1000 μm and (b) 20 μm microfluidic channels, and (c) simulated surface-bound analyte concentration profiles on the bio-receptive disc electrode surface within 1000 μm and 20 μm microfluidic channels during a 100-second injection. The color gradient represents the analyte concentration, with red indicating high concentration and blue indicating low concentration. Simulation parameters are provided in the Experimental section, ESI.†

**Fig. 3:**
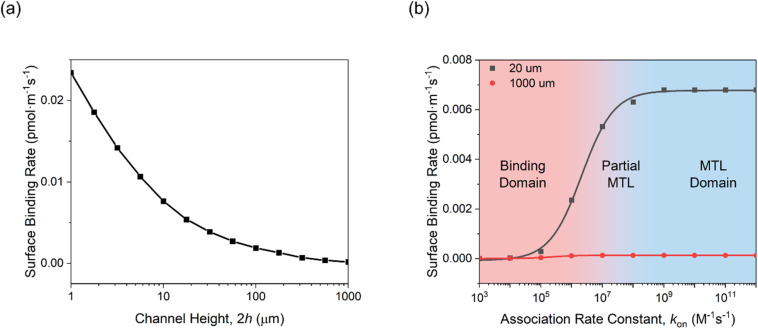
Simulation studies on the effects of (a) microfluidic channel height, and (b) association rate constant on the overall binding rate within 20 μm and 1000 μm microfluidic channels. The binding rate was determined from the first derivative of the analyte surface concentration, as resolved from the COMSOL simulation. Simulation parameters are listed in the Experimental section, ESI.†

To further validate the theoretical framework, COMSOL simulations were employed to model the effects of microfluidic confinement. Four microfluidic channel heights (20, 100, 250, and 1000 μm) were simulated to assess the impact of microfluidic confinement on mass transport and biorecognition kinetics (Fig. 2 only shows the simulated results for 1000 μm (Fig. 2a) and 20 μm (Fig. 2b) microfluidic channels; see Fig. S1† for the complete set of simulation results and simulation details in the Experimental section, ESI†). As depicted in Fig. 2c, a crescent-shape analyte surface concentration profile was noted within the 20 μm channel, consistent with expectations.^51^ This observation arises because the diffusion layer, at the upstream edge of the electrode, is vanishingly thin, allowing analyte to rapidly access the receptive surface. However, as the flow progresses downstream, analyte depletion near the surface makes the diffusion boundary gradually thickens, resulting in a lower analyte accumulation.^51^ Additionally, the surface-bound analyte concentration within the 20 μm microfluidic channel, at equilibrium, is resolved to be 30 times higher than that in a 1000 μm channel (Fig. S2, ESI†). This enhancement is accompanied by a significantly faster rate of target accumulation, as shown in Fig. 3a.

Simulations also confirmed that enhanced analyte flux is directly responsible; as shown in Fig. S3,† strong convection was noted within the 20 μm microfluidic channel, characterized by a “Levich-like” relationship (eqn (3)) when the flow rate exceeded 1 mm s^−1^ (corresponding to a Reynolds number Re = 0.1). Changes in flow dynamics, however, had limited effects in the 1000 μm channel, where diffusion is more limiting. This is because, in larger channels, the prolonged distance analytes must travel to reach the receptive surface results in the convective contribution diminishing near the surface, while axial and transverse diffusion dominate mass transport.^51^

Simulations also support the scaling of these flux enhancements with both binding affinity and bioreceptor coverage. As shown in Fig. 3b, the rate of target accumulation at the surface is much more dramatically affected by the effects of channel confinement as “association rate constant *k*_on_” grows from the nonspecific (*k*_on_ < 10^5^ M^−1^ s^−1^),^62^ to specific (*k*_on_ ≥ 10^5^ M^−1^ s^−1^)^54^ regimes and mass transport limitations become more dominant. The resulting preferential acceleration of specific over nonspecific binding is expected to improve assay selectivity. Target capture probability can also scale the enhancements (Fig. S4, ESI†). At low receptor coverage (≤10^−5^ pmol cm^−2^), capture probability is limiting with only minimal enhancement of target accumulation rate under increasing channel flux. As bioreceptor density increases, however, so the rate-limiting step transitions to mass transport and the beneficial effects of channel confinement and increased flux return (an effect scaling with receptor density until the receptive surface is antibody-saturated).

Since numerical results for microfluidic enhancements are highly dependent on key parameters (*e.g.*, flow rate, analyte concentration and diffusivity, receptive surface area, and surface-bound receptor density), to generalise these findings a dimensionless analysis was employed to demonstrate these effects are general and not peculiar to specific parameters used herein.^63^ These analyses consider two key dimensionless numbers, the Peclet number Pe and the Damköhler number Da (see Experimental section for details, ESI†). Pe (or dimensionless flow rate) quantifies the relative contribution of convection to diffusion, as shown in eqn (14).14
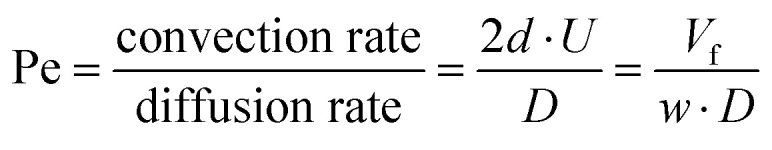
Here, 2*d* (in cm) represents the channel height, *w* is the channel width in cm, *U* is the flow velocity in cm s^−1^, *V*_f_ the volume flow rate in cm^3^ s^−1^, and *D* is the analyte diffusion coefficient in cm^2^ s^−1^. A large Peclet number indicates that convection dominates mass transport, with a small one being associated with diffusion-driven transport. As shown in Fig. S5,† the dimensionless surface binding rate exhibited a strong dependence on Pe, transitioning from a diffusion-limited regime (Pe < 10^3^) to a convection-enhanced binding regime (Pe > 10^3^), where a significant increase in dimensionless surface binding rate—normalised to receptor surface coverage *c*_B,s_, analyte bulk concentration *c*_A,b_, diffusivity *D*, and channel dimensions—was noted. Additionally, the Damköhler number Da serves as the dimensionless rate constant, defined as follows:15
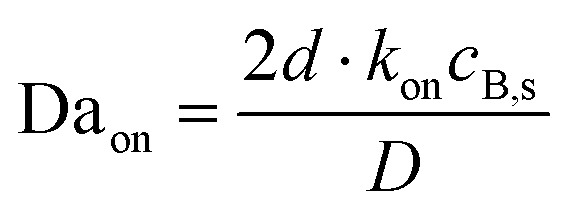


As shown in Fig. S6,† three regimes were also noted, namely those of MTL (mass-transfer limited), partial MTL, and binding regimes, aligning with the simulation results of Fig. 3b.

### Accelerated binding kinetics and increased assay sensitivity

The above discussed effects of microfluidic channel flux in enhancing key sensor characteristics were pleasingly borne out experimentally. We commenced our study of microfluidic confinement with an investigation into enhanced analyte flux within two 3D-printed channels (20 μm and 1000 μm above gold disc electrodes; two channel heights used to initially resolve diffusive effects with a redox probe; see Fig. S7 ESI† for channel fabrication details). Under a continuous flow of 5 mM ferrocyanide and ferricyanide in PBS (pH = 7.4), voltammograms obtained within the 1000 μm channel, as expected, displayed a pair of reversible redox peaks centered at *E*_half_ = +0.17 V (*vs.* Ag/AgCl) at a scan rate of 100 mV s^−1^ across all accessible flow rates (0–300 μL min^−1^).^64^ In contrast, voltammograms acquired within the 20 μm confinement demonstrated a strong dependence on flow rate. Specifically, as the flow rate increased, enhanced convective contributions meant voltammogram forms transitioned from diffusion-controlled patterns to convection-dominated profiles.^51^ The associated steady-state currents (Fig. S8, ESI†) also exhibited a dependence on flow rate, consistent with the Levich equation (eqn (3), see Fig. S9, ESI†);^51^ at identical flow rates and voltammetric scan rates, the maximum current within the 20 μm channel was significantly higher than that observed in the 1000 μm channel (42.8 ± 0.59 μA *vs.* 15.0 ± 0.27 μA), highlighting the enhanced flux supportable in the former case (at the same flow rate).

This enhanced analyte flux, of course, extends to the recruitment of macromolecular targets at the bio-receptive electrodes. Electropolymerized PANI electrodes were prepared *via* a 10-min chronopotentiometry deposition at a current density of 10 μA cm^−2^ (Fig. S10, ESI†).^48^ The resulting Fourier Transform Infrared (FT-IR) spectra, water contact angle measurements, and voltammetry scans were consistent with expectations (Fig. S11–S13, ESI†).^49^ The immobilization of anti-CRP on the PANI surface *via* glutaraldehyde cross-linking, validated using a standard SPR protocol (see Experimental section, ESI†), resulted in bio-receptive antibody coverage of approximately 189.0 ± 12.3 ng cm^−2^, equivalent to 60.0 ± 4.0% of a theoretical monolayer of IgG antibodies.^65^ This surface modification was associated with an increase in the water contact angle from 43.7° (PANI) to 56.3° (anti-CRP/PANI), and a decrease in redox capacitance *C*_r_ (see Fig. S14, ESI†), as reported by the inflection point shift in capacitive Nyquist plots.^49^

**Fig. 4:**
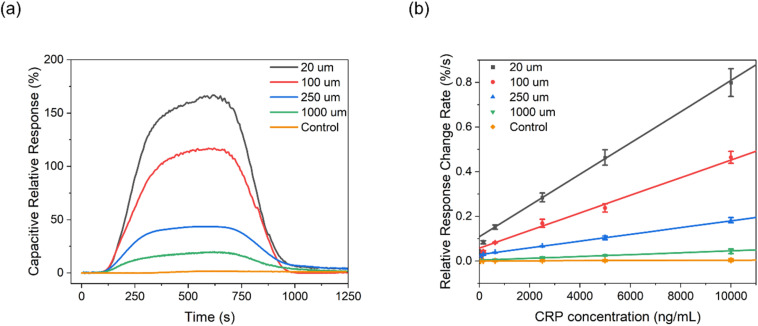
(a) Representative capacitive sensograms of the anti-CRP modified PANI interface in response to 10.0 μg mL^−1^ CRP within varying microfluidic channel heights and under a flow rate of 10 μL min^−1^. (b) Capacitive dose–response curves derived from the 15 seconds of temporal data during the association regime. *C*_r_ was normalized relative to baseline before each injection. The control (orange) sampling represents that from PANI electrodes without anti-CRP modification, measured within a 1000 μm channel. Error bars represent the standard deviation across three individual electrodes (*n* = 3).

**Fig. 5:**
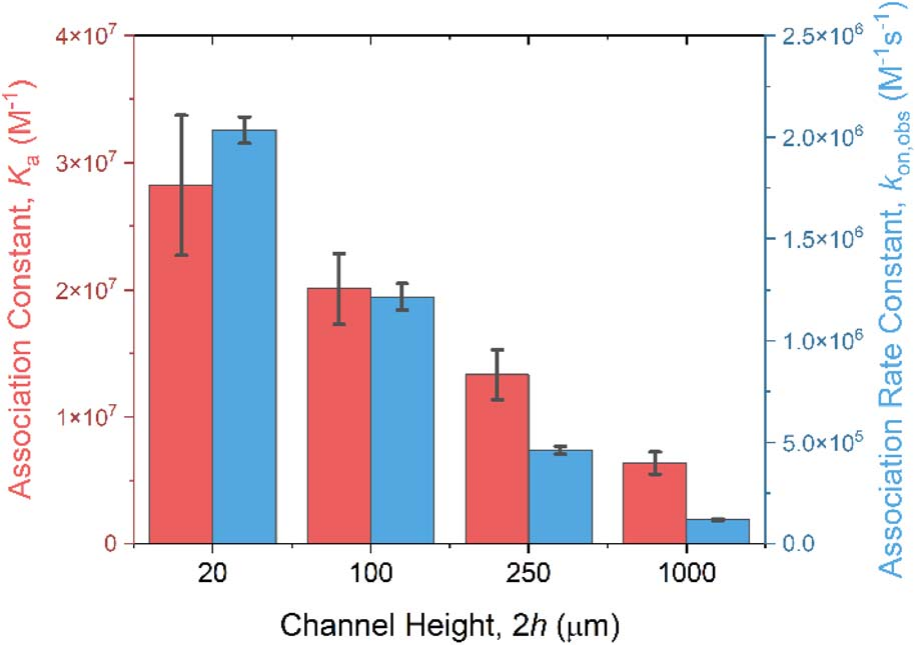
The effect of microfluidic channel height 2*h* on the association constant *K*_a_ (red) and the observed association rate constant *k*_on,obs_ (blue). *K*_a_ was determined using the Langmuir–Freundlich isotherms model by fitting equilibrium data of the capacitive response (*C*_r_ in plateau) *versus* analyte concentration (Fig. S16†). *k*_on,obs_ was derived from the first-order kinetic fitting of association *C*_r_ response, as detailed in Experimental section, ESI.†

Target CRP recruitment was performed using custom 3D-printed microfluidic cells operating at a flow rate of 10 μL min^−1^, connected to an autosampler with a sample loop volume of 100 μL (Fig. S15, ESI†). The capacitive fingerprint *C*_r_ of anti-CRP modified PANI films was monitored in real time at a fixed potential and frequency, determined from SWV and the inflection point of the capacitive Nyquist plot, respectively (see Fig. S13 and S14, ESI†), and used to transduce recruitment.^49^ As shown in Fig. 4a, a reduction in microfluidic channel height 2*h* facilitates a notably increased CRP accumulation on the PANI surface; a 10-fold increase in the normalized *C*_r_ in plateau (relative to baseline, calculated using the equation *RR*% = (1/*C*_r,t_ − 1/*C*_r,0_)/(1/*C*_r,0_) × 100) within the 20 μm channel, compared with that observed in the 1000 μm channel (167% *vs.* 19.8%) in response to 10.0 μg mL^−1^ CRP injection (see Fig. S16† for full dose–response data). Additionally, the association binding kinetics (*k*_on,obs_ evaluated directly from the maximum value of the 1st derivative of the *C*_r_ response over a 15-second sampling window)^49^ were significantly accelerated (by 2000%) in the confined channels (Fig. 4b and 5). Data analysis (see Experimental section for details, ESI†) identifies a significant increase in *k*_Lev_ in narrow channels (eqn (3)) as the origin of these observations, shifting the rate-limiting step in target surface recruitment from (sluggish two-dimensional) mass transport to binding kinetics (eqn (6)), amplifying *k*_on,obs_.

The increase in target binding kinetics necessarily leads to an increase in the thermodynamics of target association with receptive antibodies. As shown in Fig. 5, a 5-fold increase in the association constant *K*_a_ was noted upon reducing channel height from 1000 μm to 20 μm, as determined from the Langmuir–Freundlich fitting of the signal plateau (Fig. S16, ESI†). Given the relationship *K*_a_ = *k*_on,obs_/*k*_off_ (where *k*_off_ represents the dissociation rate constant), this increased binding affinity primarily results from a 20-fold increase in *k*_on,obs_*versus* a much-reduced increase in dissociation *k*_off_. As the rate constant *k*_off_ is generally significantly smaller than *k*_on_ and *k*_Lev_,^66^ the dissociation process, unlike association, remains relatively unaffected by increases in mass transport (*k*_off,obs_ = (*k*_off_^−1^ + *k*_Lev_^−1^)^−1^ ≈ *k*_off_).

The increased target binding affinity predictably supports a marked increase in assay sensitivity (Fig. S17, ESI† and Fig. 4b demonstrate a 600% in the dose–response). This enhancement, as channel height is reduced from 1000 μm to 20 μm, is accompanied by a >2 orders of magnitude reduction in the assay Limit of Detection (LoD, low picomolar, from 0.254 nM to 12.5 pM).

**Fig. 6:**
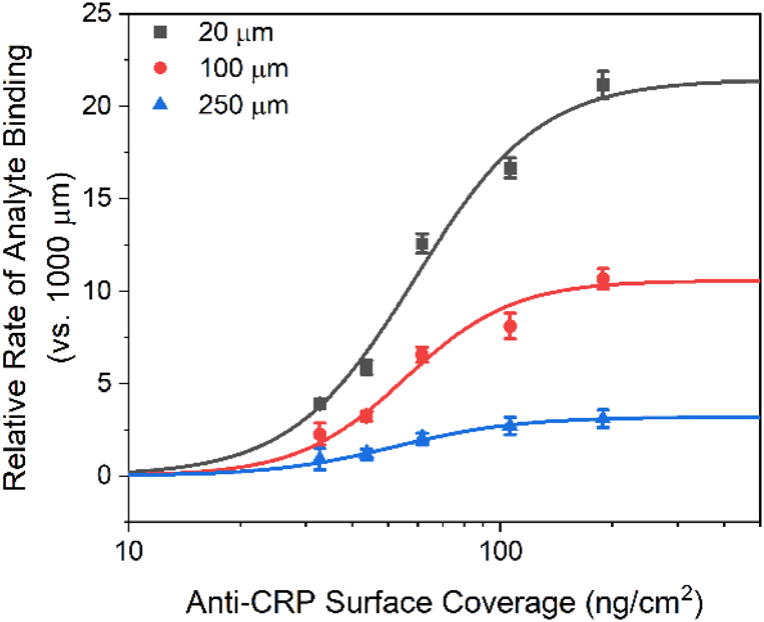
The impact of microfluidic flux enhancement as a function of (SPR determined) antibody surface density. The binding rate was normalized as the ratio of the temporal *C*_r_ change in response to 10 μg mL^−1^ CRP samples within microfluidic channels of 20, 100, and 250 μm *versus* 1000 μm height, respectively. Error bars represent the standard deviation across three individual electrodes (*n* = 3).

To further investigate the impact of microfluidic confinement on assay performance, we next examined how target capture probability—modulated through receptor density—affects the signal acquisition rate (eqn (4)). As expected, receptor density directly scales the microfluidic enhancements, as shown in Fig. 6, where two distinct regimes emerge at a threshold of 20 ng cm^−2^, aligning with the simulation results (Fig. S4, ESI†).

### Enhanced assay selectivity

**Fig. 7:**
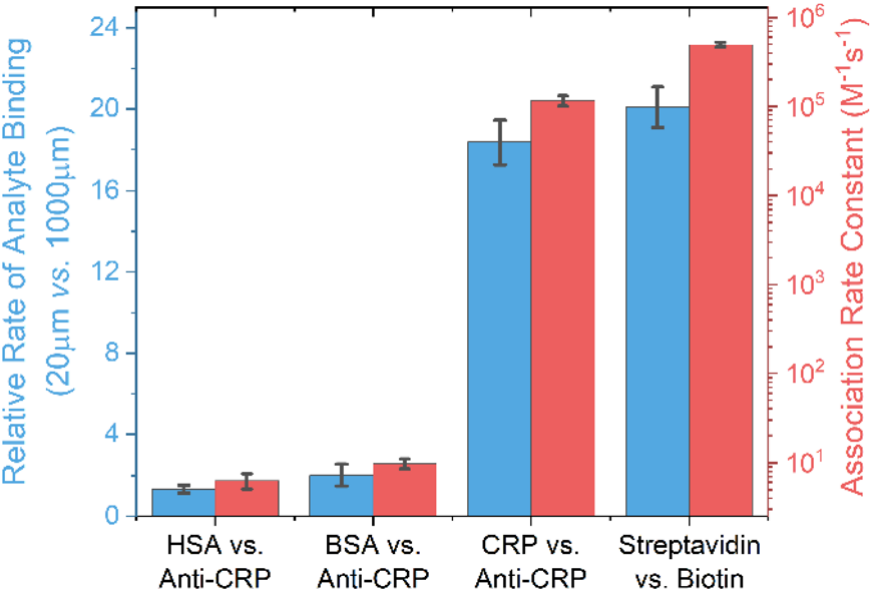
The strong resolved correlation between the enhancement of target binding rate on channel compression and the association rate constant *k*_on_ for nonspecific (HSA/anti-CRP, BSA/anti-CRP) and specific (CRP/anti-CRP, streptavidin/biotin) biorecognition pairs. Association rate constants were derived from SPR sensograms, as detailed in the Experimental section, ESI.†

**Fig. 8:**
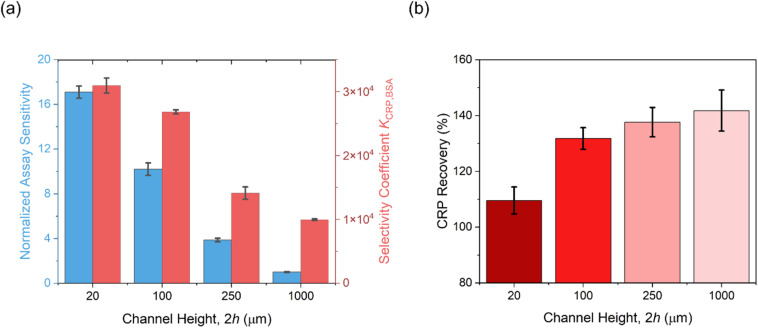
(a) The impact of microfluidic channel height on assay sensitivity and selectivity, and (b) recovery of CRP spiked into human serum. Relative sensitivity was normalized by comparing the CRP dose-temporal response slope to those observed at a channel height of 1000 μm. The selectivity coefficient was calculated as the ratio of these slopes for CRP (target) and BSA (control). CRP recovery in serum was calculated as the average across four concentrations (1.25, 2.50, 5.00, and 10.0 μg mL^−1^) at each channel height. Error bars represent the standard deviations from three independent measurements (*n* = 3).

Nonspecific fouling, arising from the undesired accumulation of background proteins and other interferents, imposes significant challenges in real-world interfacial sensing, where false positives can occur and specific target recruitment can be inhibited (necessitating antifouling strategies or surface engineering).^25,67^ We have shown above that an increase in sample flux enhances both *k*_on,obs_ and *K*_a_. These effects are, of course, expected to be much more pronounced when mass transport dominates surface accumulation—that is, when *k*_on_ is very high, as seen in specific binding events (see earlier theoretical analysis and Fig. 3b). This is indeed experimentally borne out (Fig. 7) by comparing the temporal *C*_r_ changes for specific/nonspecific biorecognition pairs, where the four orders difference in association rate constants (SPR determined; see the Experimental section, ESI†) between specific and nonspecific events enables a very substantial 300% enhancement in selectivity when flux is boosted by compressing the delivery channel (see selectivity coefficient *K*_CRP/BSA_ in Fig. 8a; data analysis is detailed in the Experimental section, ESI†). Very strong correlations (Pearson correlation coefficients of 0.999 and 0.874 respectively) were noted between this increase in specificity and the SPR-resolved association rate constant log_10_*k*_on_ and log_10_*K*_a_, respectively.

Building upon these enhanced selectivity observations, we sought to investigate the possibility of these entirely un-passivated receptive PANI electrodes to support specific target assays in serum (Fig. 8b). As expected, with 1000 μm channels, poor recoveries (>150%) were noted in serum-spiked samples particularly when CRP concentrations were below 1.0 μg mL^−1^. Attempts to mitigate nonspecific fouling by applying blocking agents (*e.g.*, SuperBlock™ blocking buffer and powdered milk), were limited. It is very notable that CRP recovery approached unity with samples spiked in diluted human serum (20 μm *vs.* 1000 μm: 109.6 ± 4.8% *vs.* 141.8 ± 7.4%), as the channel height decreased. These results highlight the microfluidic confinement in minimizing nonspecific fouling, enabling robust target detection in serum using simple, untreated, and un-passivated sensor surfaces (see Fig. S18† for dose–response curves in serum-spiked samples).

The assaying performance, as quantified by sensitivity (12 pM herein with 20 μM channels *vs.* 70 pM in previous work) and selectivity (*K*_CRP/BSA,20μm_ = 31 000 in this study *vs. K*_CRP/BSA,previous_ = 3000), is significantly enhanced compared to previous work,^49^ highlighting the potent role microfluidic confinement can have in enhancing interfacial binding.

## Conclusions

In this study, we have demonstrated the pivotal role of microfluidic confinement in enhancing the sensitivity and selectivity within a simple interfacial sensing system. By leveraging the precise fluid manipulation that microfluidics offers, we are able to significantly amplify assay response and sensitivity without any resort to conventional amplification strategies that rely on nanomaterials or catalytic processes. The integration of microfluidic configurations with electrochemical sensors has, to date, not only showcased versatility in streamlining workflow and minimizing experimental variability. We have shown here that very simple channel height engineering can support profoundly beneficial convective effects that accelerate target acquisition and response magnitudes. We have shown that these enhancements thereafter support an enhanced and very sensitive assaying of a model marker, CRP (low picomolar LoD). This work has also shown that these microfluidic enhancements preferentially favor specific binding over nonspecific fouling and this, in turn, can be leveraged to significantly increase interfacial selectivity. In leveraging these effects, we have demonstrated the assaying of a clinically relevant (CRP) marker in serum using untreated, and unprotected immunosensor surfaces, eliminating the need for pretreatment or additional blocking steps.

In combining theoretical derivations and COMSOL simulations, the underlying mechanisms driving these effects have been resolved as enhanced analyte flux within compressed microfluidic channels where mass transport limitations have been removed. These effects are, of course, most pertinent to the mass transport-limited (MTL) or partial MTL regimes of interfacial binding.^27,68,69^ These results, then, clearly highlight the potential optimization of real-world-relevant biosensor performance through (3D printable) device design. We have specifically presented a versatile, accessible, and powerful strategy for promoting enhancements in sensitivity and selectivity.

## Data availability

The data supporting this article have been included as part of the ESI,† where additional experimental detail, materials, instruments, and relevant protocols are included. It also includes the PANI interface characterization, microfluidic cell and flow system schematics, CRP recovery in serum data, the effects of channel height on binding affinity, COMSOL simulated sensograms, and simulations exploring the influence of flow rate and association rate constant on interfacial biorecognition.

## Author contributions

Electrochemical, SPR measurements, downstream electrochemical assays and COMSOL simulation were conducted by S. K. The manuscript was written by S. K., and J. J. D. J. J. D. was responsible for the concepts and experimental design. All authors have given approval to the final version of the manuscript.

## Conflicts of interest

The authors declare no competing financial interest.

## Supplementary Material

SC-016-D5SC00199D-s001

## References

[cit1] Strimbu K., Tavel J. A. (2010). What are biomarkers?. Curr. Opin. HIV AIDS.

[cit2] Aronson J. K., Ferner R. E. (2017). Biomarkers-a general review. Curr. Protoc. Pharmacol..

[cit3] Wu L., Qu X. (2015). Cancer biomarker detection: recent achievements and challenges. Chem. Soc. Rev..

[cit4] Hayes D. F. (2015). Biomarker validation and testing. Mol. Oncol..

[cit5] Grieshaber D., MacKenzie R., Vörös J., Reimhult E. (2008). Electrochemical biosensors-sensor principles and architectures. Sensors.

[cit6] Kissinger P. T. (2005). Biosensors-a perspective. Biosens. Bioelectron..

[cit7] Ronkainen N. J., Halsall H. B., Heineman W. R. (2010). Electrochemical biosensors. Chem. Soc. Rev..

[cit8] Guo L., Zhao Y., Huang Q., Huang J., Tao Y., Chen J., Li H.-Y., Liu H. (2024). Electrochemical protein biosensors for disease marker detection: progress and opportunities. Microsyst. Nanoeng..

[cit9] Sharafeldin M., Davis J. J. (2020). Point of care sensors for infectious pathogens. Anal. Chem..

[cit10] Mehrvar M., Abdi M. (2004). Recent developments, characteristics, and potential applications of electrochemical biosensors. Anal. Sci..

[cit11] Clark L. (1958). Monitoring and control blood and tissue oxygen. Trans. Am. Soc. Artif. Intern. Organs.

[cit12] Cesewski E., Johnson B. N. (2020). Electrochemical biosensors for pathogen detection. Biosens. Bioelectron..

[cit13] Amiri M., Bezaatpour A., Jafari H., Boukherroub R., Szunerits S. (2018). Electrochemical methodologies for the detection of pathogens. ACS Sens..

[cit14] Banakar M., Hamidi M., Khurshid Z., Zafar M. S., Sapkota J., Azizian R., Rokaya D. (2022). Electrochemical biosensors for pathogen detection: an updated review. Biosensors.

[cit15] Vidic J., Manzano M. (2021). Electrochemical biosensors for rapid pathogen detection. Curr. Opin. Electrochem..

[cit16] Bandodkar A. J., Wang J. (2014). Non-invasive wearable electrochemical sensors: a review. Trends Biotechnol..

[cit17] Windmiller J. R., Wang J. (2013). Wearable electrochemical sensors and biosensors: a review. Electroanalysis.

[cit18] Hernández-Rodríguez J. F., Rojas D., Escarpa A. (2020). Electrochemical sensing directions for next-generation healthcare: trends, challenges, and frontiers. Anal. Chem..

[cit19] Wu J., Liu H., Chen W., Ma B., Ju H. (2023). Device integration of electrochemical biosensors. Nat. Rev. Bioeng..

[cit20] Rodriguez-Mozaz S., de Alda M. J. L., Marco M.-P., Barceló D. (2005). Biosensors for environmental monitoring: a global perspective. Talanta.

[cit21] Hanrahan G., Patil D. G., Wang J. (2004). Electrochemical sensors for environmental monitoring: design, development and applications. J. Environ. Monit..

[cit22] Wang J. and Rogers K., Electrochemical sensors for environmental monitoring: a review of recent technology, US Environmental Protection Agency, 1995

[cit23] Justino C. I., Duarte A. C., Rocha-Santos T. A. (2017). Recent progress in biosensors for environmental monitoring: a review. Sensors.

[cit24] Chu N., Liang Q., Hao W., Jiang Y., Liang P., Zeng R. J. (2021). Microbial electrochemical sensor for water biotoxicity monitoring. Chem. Eng. J..

[cit25] Lin P.-H., Li B.-R. (2020). Antifouling strategies in advanced electrochemical sensors and biosensors. Analyst.

[cit26] Wu Y., Tilley R. D., Gooding J. J. (2018). Challenges and solutions in developing ultrasensitive biosensors. J. Am. Chem. Soc..

[cit27] Gervais T., Jensen K. F. (2006). Mass transport and surface reactions in microfluidic systems. Chem. Eng. Sci..

[cit28] Cho I.-H., Lee J., Kim J., Kang M.-s., Paik J. K., Ku S., Cho H.-M., Irudayaraj J., Kim D.-H. (2018). Current technologies of electrochemical immunosensors: Perspective on signal amplification. Sensors.

[cit29] Lee T. M.-H., Carles M. C., Hsing I.-M. (2003). Microfabricated PCR-electrochemical device for simultaneous DNA amplification and detection. Lab Chip.

[cit30] Lim S. A., Ahmed M. U. (2016). Electrochemical immunosensors and their recent nanomaterial-based signal amplification strategies: a review. RSC Adv..

[cit31] Wang Z., Dai Z. (2015). Carbon nanomaterial-based electrochemical biosensors: an overview. Nanoscale.

[cit32] Malhotra B. D. and Ali M. A., Nanomaterials in biosensors: Fundamentals and applications, Elsevier, Amsterdam, 2018

[cit33] Holzinger M., Le Goff A., Cosnier S. (2014). Nanomaterials for biosensing applications: a review. Front. Chem..

[cit34] Li L., Wang T., Zhong Y., Li R., Deng W., Xiao X., Xu Y., Zhang J., Hu X., Wang Y. (2024). A review of nanomaterials for biosensing applications. J. Mater. Chem. B.

[cit35] Bahadır E. B., Sezgintürk M. K. (2016). Applications of graphene in electrochemical sensing and biosensing. TrAC, Trends Anal. Chem..

[cit36] Arya S. K., Estrela P. (2018). Electrochemical ELISA-based platform for bladder cancer protein biomarker detection in urine. Biosens. Bioelectron..

[cit37] Arya S. K., Estrela P. (2018). Recent advances in enhancement strategies for electrochemical ELISA-based immunoassays for cancer biomarker detection. Sensors.

[cit38] Hosseini S. , Vázquez-Villegas P., Rito-Palomares M. and Martinez-Chapa S. O., in Enzyme-Linked Immunosorbent Assay (ELISA), Springer, 2018, pp. 67–115

[cit39] Sharafeldin M., Fleschhut F., James T., Davis J. J. (2022). A quantification of target protein biomarkers in complex media by faradaic shotgun tagging. Anal. Chem..

[cit40] Sharafeldin M., Yan S., Jiang C., Tofaris G. K., Davis J. J. (2023). Alternating magnetic field-promoted nanoparticle mixing: the on-chip immunocapture of serum neuronal exosomes for Parkinson's disease diagnostics. Anal. Chem..

[cit41] Kulkarni M. B., Ayachit N. H., Aminabhavi T. M. (2022). Biosensors and microfluidic biosensors: from fabrication to application. Biosensors.

[cit42] Muhsin S. A., He Y., Al-Amidie M., Sergovia K., Abdullah A., Wang Y., Alkorjia O., Hulsey R. A., Hunter G. L., Erdal Z. K. (2023). A microfluidic biosensor architecture for the rapid detection of COVID-19. Anal. Chim. Acta.

[cit43] Ehrnström R. (2002). Profile: Miniaturization and integration: challenges and breakthroughs in microfluidics. Lab Chip.

[cit44] Lion N., Reymond F., Girault H. H., Rossier J. S. (2004). Why the move to microfluidics for protein analysis?. Curr. Opin. Biotechnol..

[cit45] Rackus D. G., Shamsi M. H., Wheeler A. R. (2015). Electrochemistry, biosensors and microfluidics: a convergence of fields. Chem. Soc. Rev..

[cit46] Fernández-la-Villa A., Pozo-Ayuso D. F., Castaño-Álvarez M. (2019). Microfluidics and electrochemistry: an emerging tandem for next-generation analytical microsystems. Curr. Opin. Electrochem..

[cit47] Lafleur J. P., Jönsson A., Senkbeil S., Kutter J. P. (2016). Recent advances in lab-on-a-chip for biosensing applications. Biosens. Bioelectron..

[cit48] Baradoke A., Hein R., Li X., Davis J. J. (2020). Reagentless redox capacitive assaying of C-reactive protein at a polyaniline interface. Anal. Chem..

[cit49] Kang S., Sharafeldin M., Patrick S. C., Chen X., Davis J. J. (2023). Ultrafast biomarker quantification through reagentless capacitive kinetics. Anal. Chem..

[cit50] Cooper J. A., Compton R. G. (1998). Channel electrodes-a review. Electroanalysis.

[cit51] Compton R. G. and Banks C. E., Understanding Voltammetry, World Scientific, 3rd edn, 2018

[cit52] Barr D. I. H. (1980). The Transition from Laminar to Turbulent Flow. Proc. Instn. Civ. Engrs..

[cit53] Northrup S. H., Erickson H. P. (1992). Kinetics of protein-protein association explained by Brownian dynamics computer simulation. Proc. Natl. Acad. Sci. U. S. A..

[cit54] Duan X., Li Y., Rajan N. K., Routenberg D. A., Modis Y., Reed M. A. (2012). Quantification of the affinities and kinetics of protein interactions using silicon nanowire biosensors. Nat. Nanotechnol..

[cit55] Dejardin P., Le M., Johner A., Wittmer J. (1994). Adsorption rate in the convection-diffusion model. Langmuir.

[cit56] Déjardin P., Vasina E. N. (2004). An accurate simplified data treatment for the initial adsorption kinetics in conditions of laminar convection in a slit: application to protein adsorption. Colloids Surf., B.

[cit57] Li B., Chen J., Long M. (2008). Measuring binding kinetics of surface-bound molecules using the surface plasmon resonance technique. Anal. Biochem..

[cit58] Rabbany S. Y., Kusterbeck A. W., Bredehorst R., Ligler F. S. (1995). Binding kinetics of immobilized antibodies in a flow immunosensor. Sens. Actuators, B.

[cit59] Hu G., Gao Y., Li D. (2007). Modeling micropatterned antigen-antibody binding kinetics in a microfluidic chip. Biosens. Bioelectron..

[cit60] Skopp J. (2009). Derivation of the Freundlich adsorption isotherm from kinetics. J. Chem. Educ..

[cit61] Harvey D. , Modern Analytical Chemistry, McGraw-Hill, New York, 2000

[cit62] Ogi H., Fukunishi Y., Nagai H., Okamoto K., Hirao M., Nishiyama M. (2009). Nonspecific-adsorption behavior of polyethylenglycol and bovine serum albumin studied by 55-MHz wireless–electrodeless quartz crystal microbalance. Biosens. Bioelectron..

[cit63] Squires T. M., Messinger R. J., Manalis S. R. (2008). Making it stick: convection, reaction and diffusion in surface-based biosensors. Nat. Biotechnol..

[cit64] Bard A. J. , Faulkner L. R. and White H. S., Electrochemical Methods: Fundamentals and Applications, Wiley, Hoboken, 3rd edn, 2022

[cit65] Macchia E., Manoli K., Holzer B., Di Franco C., Picca R. A., Cioffi N., Scamarcio G., Palazzo G., Torsi L. (2019). Selective single-molecule analytical detection of C-reactive protein in saliva with an organic transistor. Anal. Bioanal. Chem..

[cit66] Schreiber G., Haran G., Zhou H.-X. (2009). Fundamental aspects of protein-protein association kinetics. Chem. Rev..

[cit67] Jiang C., Wang G., Hein R., Liu N., Luo X., Davis J. J. (2020). Antifouling strategies for selective *in vitro* and *in vivo* sensing. Chem. Rev..

[cit68] Sigmundsson K., Másson G., Rice R., Beauchemin N., Öbrink B. (2002). Determination of active concentrations and association and dissociation rate constants of interacting biomolecules: an analytical solution to the theory for kinetic and mass transport limitations in biosensor technology and its experimental verification. Biochemistry.

[cit69] Christensen L. L. (1997). Theoretical analysis of protein concentration determination using biosensor technology under conditions of partial mass transport limitation. Anal. Biochem..

