# Supplementary material for: Leveraging microfluidic confinement to boost assay sensitivity and selectivity
Source: Chem Sci. 2025 Mar 11;16(16):6965–74. doi: 10.1039/d5sc00199d (PMC11931431; doi:10.1039/d5sc00199d)
Supplement: SC-016-D5SC00199D-s001 [file SC-016-D5SC00199D-s001.pdf]

Supplementary Information (SI) for Chemical Science.
This journal is © The Royal Society of Chemistry 2025

SUPPORTING INFORMATION

Leveraging Microfluidic Confinement to Boost Assay Sensitivity and Selectivity

Shaoyu Kang, Jason J. Davis*

* Corresponding author

Department of Chemistry, University of Oxford, South Parks Road, Oxford, OX1 3QZ, U.K.

Table of Contents

Experimental Sections S-2
Figure S1. Simulated analyte surface concentration profiles S-5
Figure S2. Simulated sensogram within microfluidic channels S-5
Figure S3. Simulation studies of flow rate effect S-6
Figure S4. Simulation studies of surface-bound receptor coverage effect S-6
Figure S5. Dimensionless analysis of the Peclet number effect S-7
Figure S6. Dimensionless analysis of the Damköhler number effect S-7
Figure S7. Schematic representation of the custom 3D-printed microfluidic channel fabrication S-8
Figure S8. Representative cyclic voltammograms within microfluidic channels at varying flow rates S-8
Figure S9. The Levich dependence between steady-state currents and flow rate S-9
Figure S10. Representative electrodeposition of PANI on Au disc electrodes S-9
Figure S11. FT-IR spectrum of the PANI-coated gold chip S-10
Figure S12. Representative water contact angles during PANI preparation S-10
Figure S13. Representative SWV scan of PANI films S-11
Figure S14. Capacitive Nyquist plots during PANI preparation S-11
Figure S15. Schematic depiction of the continuous flow microfluidic configuration S-12
Figure S16. Langmuir isotherms fitting of equilibrium redox capacitance across varying channel height S-12
Figure S17. Slope of “Dose-Response” curves as a function of microfluidic channel height S-13
Figure S18. “Dose-Response” curves in serum-spiked samples within microfluidic channels S-13
Reference S-13

EXPERIMENTAL SECTIONS

Materials and Instruments

Sodium phosphate dibasic, sodium phosphate monobasic, phytic acid solution (50% w/w in H_2O), aniline, glutaraldehyde solution (25% in water), albumin from human serum (HSA), IgG (from bovine serum, ≥ 95%), and bovine serum albumin (BSA) were purchased from Sigma (UK). Biotin-BSA, Streptavidin, Native human C-Reactive protein (CRP) and Goat anti-CRP were obtained from Bio-Rad Laboratories, Inc. (UK). All chemicals were used without further purification.

Fourier transform infrared spectra (FT-IR) were acquired using an FT-IR spectrophotometer (IRTracer-100, Shimadzu, Japan). Surface plasmon resonance (SPR) measurements were performed with a Biacore SPR instrument (Biacore X100, Cytiva). Water contact angles were measured using a goniometer (FTA1000B, First Ten Angstroms, Inc.). The continuous flow setup consisted of a custom 3D-printed microfluidic chip (fabricated with an ELEGOO Mars UV photocuring 3D printer, ELEGOO, Inc.), with channel heights above the working electrode varying from 20 μm to 1000 μm), an autosampler, and a 3-electrode electrochemical system (details provided below).

All electrochemical experiments were performed using a PalmSens potentiostat (PalmSens BV) with a three-electrode system consisting of a gold (Au) disc working electrode (ϕ = 1.6 mm, BASi), a platinum wire (Pt) counter electrode (ϕ = 0.25 mm), and a silver/silver chloride wire (Ag/AgCl) pseudo reference electrode (ϕ = 0.25 mm). The Au disc working electrodes were cleaned as follows: electrodes were first mechanically polished using a slurry of MicroPolish alumina powder (Buehler) with decreasing particle sizes (1.0, 0.3, and 0.05 μm, in sequence). After polishing, the electrodes were sonicated three times in a 1:1 mixture of deionized water and ethanol, rinsed with deionized water, and then immersed in piranha solution (*conc.* H_2SO_4 / 30% H_2O_2, *v/v* 3:1) for approximately 15 minutes. Following this step, they were thoroughly rinsed with deionized water and electrochemically polished. Electrochemical polishing was performed first in 0.5 M KOH solution using cyclic voltammetry with repeated scans from - 0.7 V to - 1.7 V (*vs.* Ag/AgCl), followed by polishing in 0.5 M H_2SO_4 solution from - 0.15 V to + 1.35 V (*vs.* Ag/AgCl) with a scan rate of 0.1 $V \cdot s^{-1}$. Both electrochemical polishing steps were carried out for approximately one hour.

Electrochemical measurements were conducted in 0.1 M PB buffer (pH 7.4), unless otherwise specified. Electrochemical impedance spectroscopy (EIS) was performed over a range of 40 frequencies from 100 kHz to 0.1 Hz using a sinusoidal AC perturbation of 5 mV, with the DC potential fixed at the half-wave potential of polyaniline (- 0.16 V, *vs.* Ag/AgCl). The capacitance of the interface was calculated using the equations $C^{'} = Z^{''}/\omega Z^2$ and $C^{''} = Z^{'}/\omega Z^2$, where ω is the angular frequencies and Z is the impedance of the interface. The redox capacitance C_r was derived from the diameter of the semicircle or the inflection point in the capacitive Nyquist plot. Square-wave voltammetry (SWV) was performed from -0.3 V to 0 V (*vs.* Ag/AgCl) with an amplitude of 20 mV, a potential step of 5 mV and a frequency of 50 Hz in 0.1 M PB buffer at a pH of 7.4.

Bio-receptive Interface Preparation

The anti-CRP/PANI interface was prepared through an electropolymerization process,[1, 2] shown as follows: Electrodeposition of polyaniline (PANI) was carried out on gold (Au) disc electrodes via chronopotentiometry, by immersing them in an aniline solution consisting of 1 mL of 98% aniline, 2 mL of 50% phytic acid, and 17 mL of deionized water, at a constant current density of 10 $\mu A \cdot cm^{-2}$ for 10 minutes. Following electrodeposition, the PANI-coated electrodes were thoroughly rinsed with 0.1 M PB buffer to remove any residual reactants. Electrodes were then activated by incubation in a 2.5% glutaraldehyde solution (prepared in 0.1 M PB) for 30 minutes at room temperature. After glutaraldehyde activation, the electrodes were incubated with a 100 $\mu g \cdot mL^{-1}$ solution of polyclonal anti-CRP antibodies for 18 hours to facilitate antibody immobilization. Finally, the electrodes were washed

thoroughly and stored in 0.1 M PB buffer until use.

Continuous Flow Assays

The prepared anti-CRP/PANI electrodes were integrated into custom-designed 3D-printed microfluidic cells (see Fig. **S7** for fabrication detail). Briefly, the assembled microfluidic channels were designed in Fusion 360, with adjustable channel heights from 20 to 1000 μm, measured from the top surface of the microchannel to the bottom surface attached to the electrode. After fabrication using stereolithography (SLA) 3D printing (ELEGOO Mars, standard resin, 5 μm layer thickness), the printed structures were post-processed by washing in isopropanol and cured under UV light to enhance mechanical stability. For assembly, Pt and Ag/AgCl wires were carefully integrated into the cells and securely glued at the inlet and outlet (Fig. **S7**), respectively, to establish a three-electrode system with PANI-coated gold disc electrodes. The assembled microfluidic cells were incorporated into a continuous flow system (Fig. **S9**), comprising an automated syringe pump equipped with a 250 μL tricontinent syringe (Reichert, Inc.) and an autosampler (SR8100, Reichert, Inc.) with a 100 μL sample loop.

Real-time redox capacitance (C_r) measurements were performed at the half-wave potential of PANI ($E_{1/2}$ = - 0.16 V *vs.* Ag/AgCl) using a fixed frequency determined from the inflection point of the capacitive Nyquist plot.[2] A stable baseline was established at a flow rate of 10 $\mu L \cdot min^{-1}$ before measurements, with a drift of less than 1% over a 10-minute period. Subsequently, analytes were introduced into the system via the autosampler, followed by a washing step with 0.1 M PB buffer to remove unbound molecules. The washing phase, lasting 30 minutes, ensured the baseline was re-established prior to subsequent analyte injections.

Data analyses were conducted using Origin2023 software. Real-time redox capacitance data were converted to relative response values using the formula: $RR\% = (1/C_{r,t} - 1/C_{r,0})/(1/C_{r,0}) \cdot 100$), where $C_{r,t}$ represents the redox capacitance at a given time, and $C_{r,0}$ is the initial redox capacitance. All data were normalized to the baseline re-established before each sample injection. The equilibrium response (C_r) at the plateau following analyte injection was used to construct "dose-response" curves. Binding constants (K_a) for biorecognition events were estimated by fitting these curves to the Langmuir isotherm. The association kinetics were evaluated by analyzing the rate of change in the relative response, which was derived directly from the first derivative of the capacitive relative response. The maximum value of this derivative represented the initial binding rate, which was then used to construct "dose-temporal response" curves. From these curves, the observed association rate constants ($k_{on,obs}$) for biorecognition pairs were determined as the slopes. To assess selectivity, the selectivity coefficient ($K_{A,I}$) for the analyte (A) over background (I) was calculated as the ratio of the slopes of their respective temporal response, normalized to their concentrations.

SPR study

Preparation and functionalization of SPR gold chips (SIA Kit Au, Cytiva) were conducted as follows: The gold chips were thoroughly rinsed with ethanol and deionized water multiple times, followed by immersion in piranha acid (composition described above) for 10 minutes to ensure a clean and reactive surface. After cleaning, the chips were coated with PANI films as described previously.[2] Antibody immobilization onto the PANI-coated chips was performed in situ using the SPR spectrometer. The chips were flushed with 0.1 M PB buffer at a flow rate of 30 $\mu L \cdot min^{-1}$ at room temperature to stabilize the baseline. They were then incubated under flow with 2.5% glutaraldehyde (prepared in PB buffer) for 30 minutes to activate the PANI surface. Following activation, the chips were exposed to a 100 $\mu g \cdot mL^{-1}$ anti-CRP antibody solution and allowed to incubate for 30 minutes to facilitate immobilization. Antibody coverage (C_s) on the chip surface was estimated using the standard Biacore working protocol, calculated as: C_s = 1.0 $\cdot \Delta RU\ pg \cdot mm^{-2}$. Here, ΔRU represents the difference in response units between the sample-injected channel and

the control channel (injected with PB buffer only). A full SPR sensogram was constructed by injecting a series of recombinant CRP solutions at increasing concentrations (from 39.1 $ng \cdot mL^{-1}$ to 10.0 $\mu g \cdot mL^{-1}$), with the association and dissociation regimes lasting 270 s and 600 s, respectively. Association rate constant k_{on} was derived from the slope of "observed rate constant (k_{obs}) *vs.* analyte concentration" plot or directly obtained from the SPR software using the Langmuir 1:1 binding model. It is noted that the observed association rate constant of streptavidin/biotin determined from SPR was two orders of magnitude lower than the reported value ($k_{on} \sim 10^7\ M^{-1}s^{-1}$),[3] potentially due to mass transport limitations under flow conditions.

COMSOL Simulation

The mass transport and surface biorecognition processes within microfluidics channels were modeled using COMSOL Multiphysics. The simulation parameters were defined as follows: The diffusion coefficient of the analyte was set to 10^{-11} $m^2 \cdot s^{-1}$, and the bulk analyte concentration was 10^{-4} $mol \cdot m^{-3}$. The surface coverage of the receptor ranged from 10^{-6} to 10^{2} $pmol \cdot cm^{-2}$, while the infused flow rate was fixed at 0.02 $cm \cdot s^{-1}$. The association rate constants were varied from 10^{3} to 10^{12} $M^{-1} \cdot s^{-1}$, and the dissociation rate constant was set at 1 s^{-1}. The parameter ranges were chosen based on our experimental results. The simulations employed the modules for Surface Reactions, Transport of Diluted Species, and Laminar Flow, with a total simulation time of 100 seconds. Analyte surface concentration profiles were computed and mathematically integrated over the surface, followed by differentiation with respect to time to determine the initial binding rate (*i.e.*, temporal response) under various conditions (*e.g.*, microfluidic channel height, flow rate, kinetic rate constants, and receptor surface coverage).

To perform dimensionless analysis in COMSOL Multiphysics, variables (*e.g.*, dimension of channel, analyte concentration in bulk concentration, diffusion coefficient, surface coverage of receptor, flow rate, and simulation timescale) must first be non-dimensionalised,[4] and relevant dimensionless parameters introduced. Herein these were the Peclet number (Pe_H, dimensionless flow rate, as defined in eq.**14**) quantifying the relative importance of convective transport and the Damköhler number (Da, dimensionless rate constant, as defined in eq.**15**, for comparing the reaction (analyte surface binding) rate to the diffusion rate. In COMSOL, the model was scaled relative to the characteristic length — that is, channel height. Dimensionless analyte surface concentration profiles were computed and mathematically integrated over the surface, as described earlier (see previous paragraph). Parametric sweeps in the COMSOL study toolbar were employed to scan the impact of dimensionless parameters across a range of Pe_H and Da_{on} values, and their impact on the dimensionless surface binding rate was systematically analysed (Fig **S5** and **S6**).

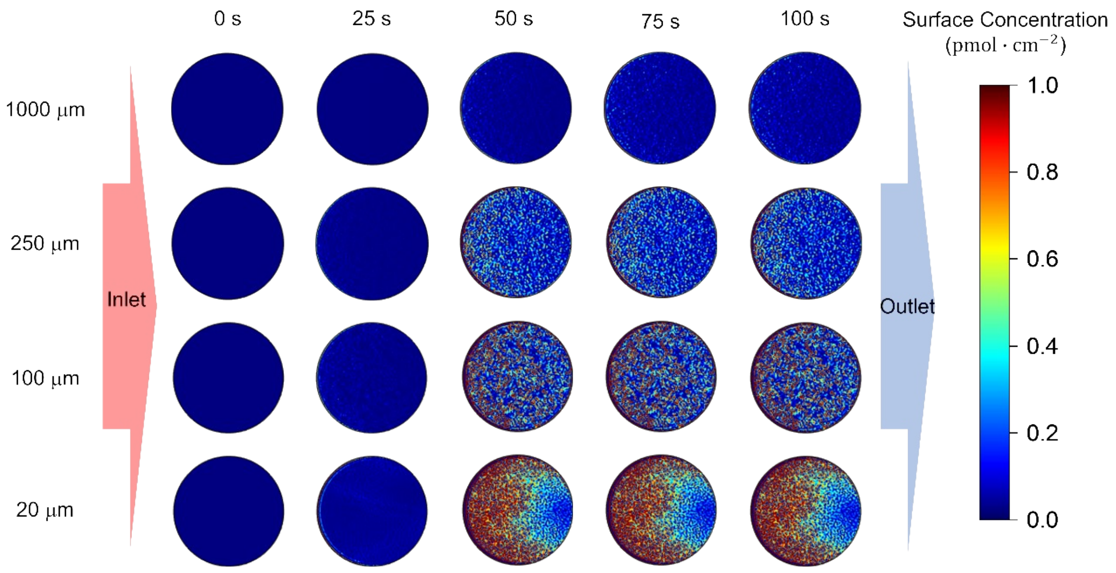

Fig. S1 Simulated temporal surface concentration profiles for 20 μm to 1000 μm microfluidic channels illustrating enhanced analyte accumulation / accelerated binding kinetics for smaller channel heights. The parameters used for the simulation, including diffusion coefficient, flow rate, and kinetic constants, are detailed in the COMSOL Simulation of the Experimental Section (Page S4).

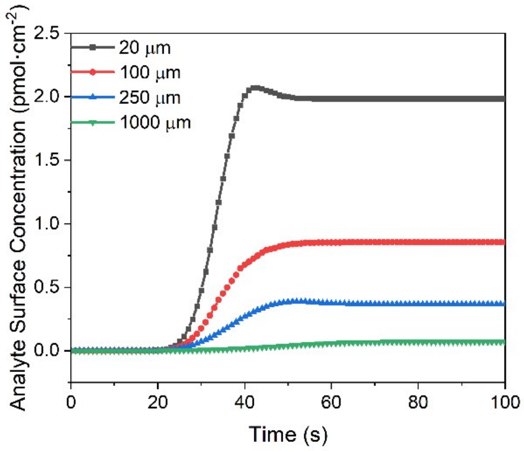

Fig. S2 Simulated sensogram of interfacial analyte binding within microfluidic channels with heights of 20, 100, 250, and 1000 μm, highlighting the enhanced analyte accumulation (saturation end-points) and accelerated binding kinetics (association phase slope) with smaller channel heights. The parameters used for the simulation, including diffusion coefficient, flow rate, and kinetic constants, are detailed in the COMSOL Simulation of the Experimental Section (Page S4).

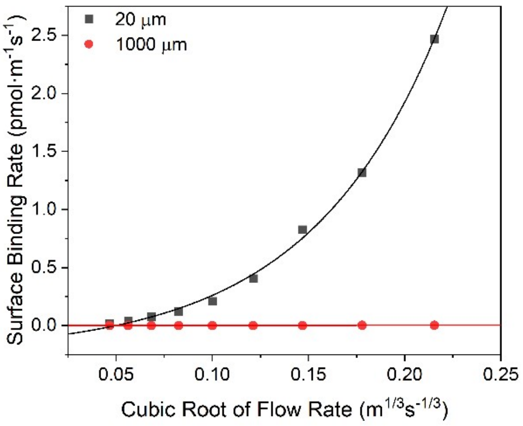

Fig. S3 Simulation studies examining the effect of flow rate on analyte binding kinetics within 20 μm (black) and 1000 μm (red) microfluidic channels. The results demonstrate a significantly increased binding rate in the 20 μm channel, with a strong dependence on flow dynamics. In contrast, the binding rate in the 1000 μm microfluidic channel is comparatively insensitive to flow rate. The binding rate was derived from the first derivative of the analyte surface concentration, as obtained from COMSOL simulations.

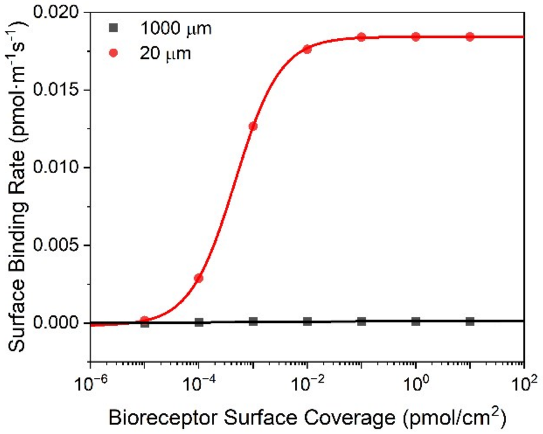

Fig. S4 Simulation studies examining the effect of receptor density on analyte binding kinetics within 20 μm (red) and 1000 μm (black) channels. It is obvious that higher bioreceptor densities amplify the effects of microfluidic enhancement. The binding rate was determined from the first derivative of the analyte surface concentration, as obtained from COMSOL simulations.

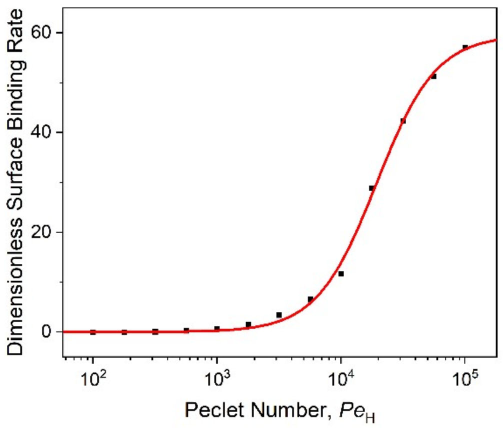

Fig. S5 Dimensionless surface binding rate as a function of the Peclet number (Pe_H, as defined in eq.**14**), showing a transition from a diffusion-limited regime ($Pe_H \leq$ 10³) to a convection-enhanced binding regime ($Pe_H \geq$ 10³). As convective transport begins to dominate over diffusion, a significant increase in surface binding efficiency is observed, resulting in enhanced analyte capture at higher flow rates.

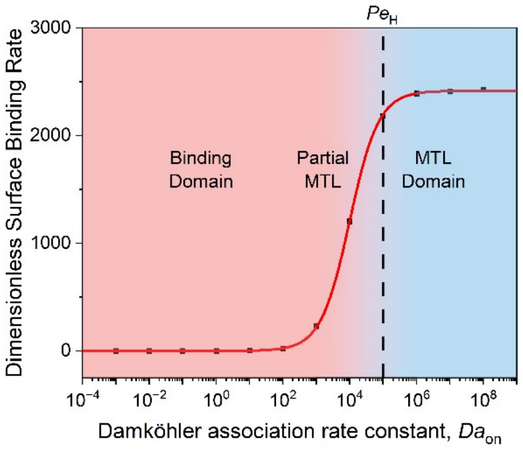

Fig. S6 Dimensionless surface binding rate as a function of the Damköhler association rate constant (Da_{on}, as defined in eq.**15**) from the binding domain (reaction-limited), partial mass transport-limited (partial MTL), to MTL domain. The transition occurs near the characteristic Peclet number (Pe_H, dashed line), indicating the onset of mass transport dominance in analyte capture.

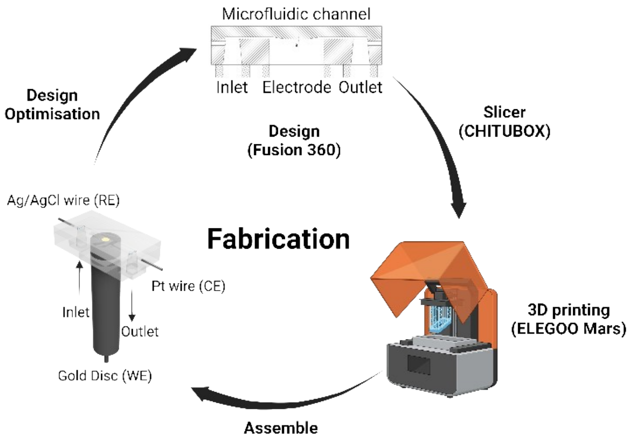

Fig. S7 Schematic representation of the fabrication workflow for custom 3D-printed microfluidic channels, from design to final functional integration, as detailed in the Continuous Flow Assays of the Experimental Section. The microfluidic channels were designed in Fusion 360 with adjustable channel heights (20 -1000 μm) and fabricated using stereolithography 3D printing (ELEGOO Mars, 5 μm layer thickness). After post-processing treatment (isopropanol washing and UV curing), Pt and Ag/AgCl wires were integrated into the microfluidic cells and securely affixed to establish a three-electrode system with gold disc electrodes. Iterative design optimization was performed to ensure precise microchannel dimensions and reliable device performance.

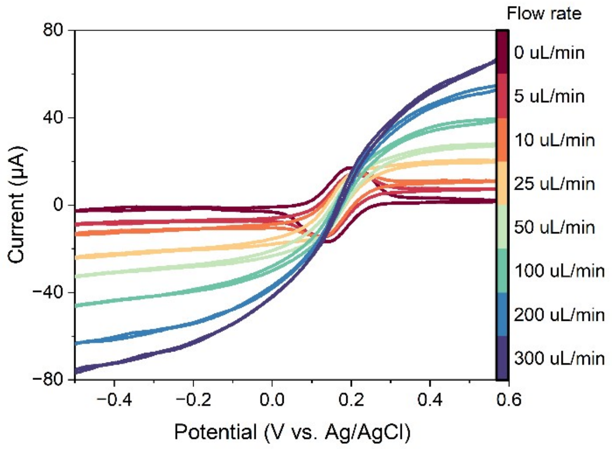

Fig. S8 The example of cyclic voltammograms within microfluidic channel (100 μm) at varying flow rates (0 - 300 $\mu L \cdot min^{-1}$) with a scan rate of 100 $mV \cdot s^{-1}$, using a 5 mM ferrocyanide/ferricyanide PB solution (pH = 7.4). As the flow rate increased, the system shifted from a diffusion-controlled to a convection-dominated regime, with steady-state currents noted at high overpotentials ($\eta \gg 0\ V$).

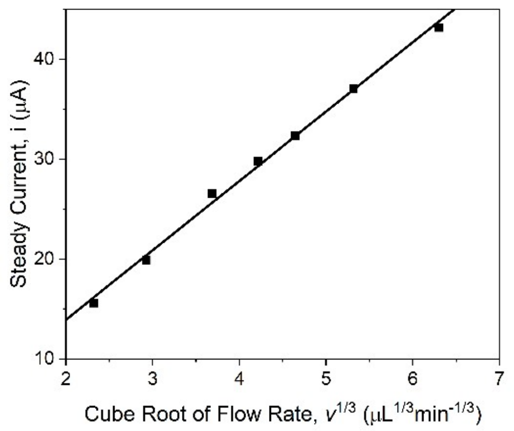

Fig. S9 The examples of Levich dependence between steady-state currents and flow rate (accessible from 5.0 $\mu L \cdot min^{-1}$ to 300 $\mu L \cdot min^{-1}$) in the 100 μm microfluidic channel under a scan rate of 100 $mV \cdot s^{-1}$, demonstrating convection-dominated mass transport behavior (eq. **3**). Steady-state currents were obtained from the cyclic voltammograms (Fig. **S2**, ESI) at a potential of + 0.60 V (vs. Ag/AgCl) using a 5 mM ferrocyanide/ferricyanide PB solution (pH = 7.4).

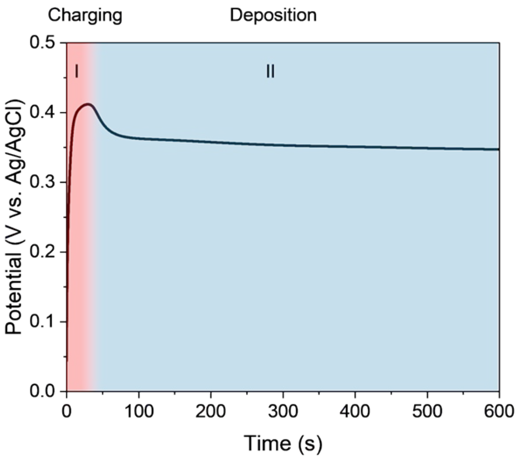

Fig. S10 Representative electrodeposition of PANI on Au disc electrodes via a 10-min chronopotentiometric scan at a current density of 10 $\mu A \cdot cm^{-2}$. An initial sharp increase in potential observed during the electrodeposition process (Region I) is attributed to double layer charging. This increase continues until the potential reaches the deposition threshold (E_{dep} = + 0.41 V *vs.* Ag/AgCl), initiating the polymerization of aniline and promoting uniform PANI film growth. The electrodeposition of conductive PANI onto the electrode surface enhances the interface capacitance, resulting in a gradual decrease in potential observed in Region II. Notably, the double-layer charging, as observed in Region I, is fundamentally different from the capacitive transducing signal (redox capacitance, C_r) used in biosensing.

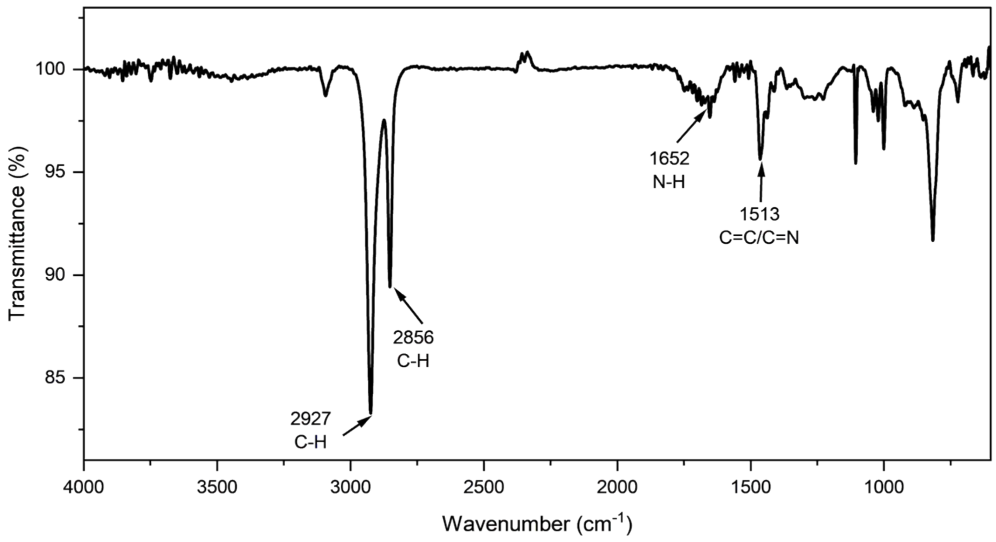

Fig. S11 FT-IR spectrum of the PANI-coated gold chip, including two sharp peaks corresponding to C-H stretching vibrations at 2927 and 2856 cm^{-1}, a peak of N-H bending at 1652 cm^{-1}, two sharp peaks of quinoid/benzenoid C=C stretching at around 1513 cm^{-1}.

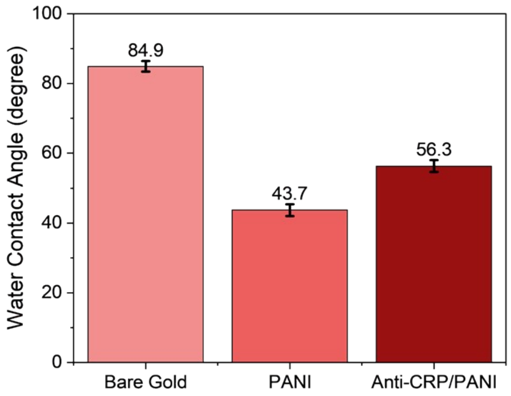

Fig. S12 Representative water contact angles of bare gold disc electrode, electro-grafted PANI films, and anti-CRP modified PANI interfaces. Error bars represent the standard deviation from three individual electrodes (n = 3).

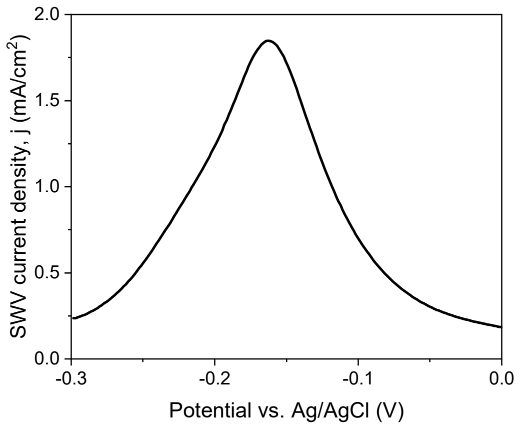

Fig. S13 Representative PANI film square wave voltammetry (SWV) fingerprints. SWV was conducted from -0.3 V to 0 V (*vs.* Ag/AgCl) with an amplitude of 20 mV, a potential step of 5 mV and a frequency of 50 Hz in 0.1 M PB buffer, pH of 7.4.

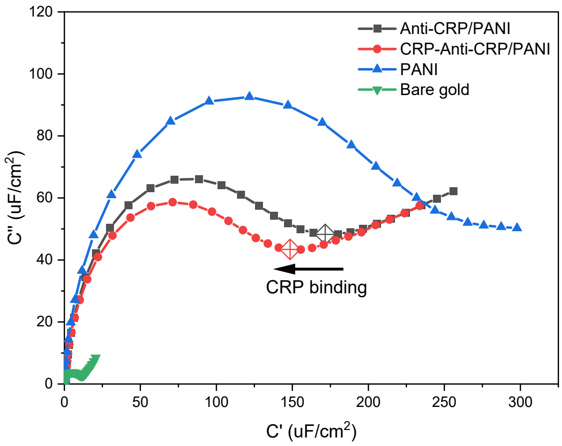

Fig. S14 Capacitive Nyquist plots of a bare gold electrode (green), PANI film (blue), and anti-CRP/PANI interface before (black) and after (red) CRP injection. Capacitance was measured in 0.1 M PB buffer (pH 7.4) at a potential of E_{half} = -0.16 V *vs.* Ag/AgCl. The inflection points labelled with rhombus indicate the redox capacitance of the PANI interface.

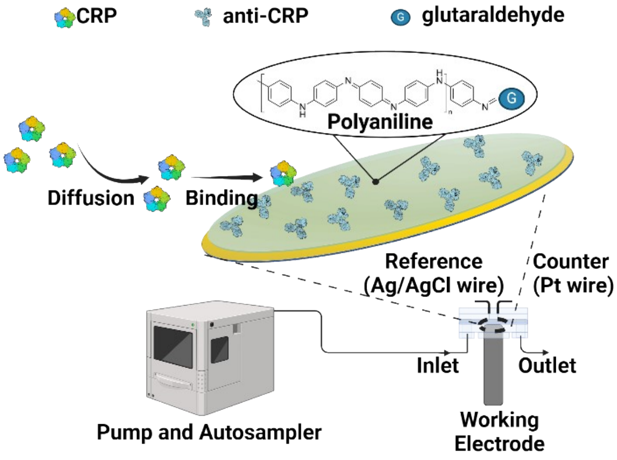

Fig. S15 Schematic depiction of the continuous flow microfluidic configuration housing an anti-CRP/PANI modified Au disc electrode (ϕ = 1.6 mm) for electrochemical capacitance spectroscopy (ECS) assays. The electrode is inserted into a custom 3D-printed microfluidic cell with a Ag/AgCl wire reference electrode (ϕ = 0.25 mm) and Pt wire counter electrode (ϕ = 0.25 mm). The sample and running buffer are delivered by an automated syringe pump and autosampler (see the Experimental Section, ESI), with a sample loop volume of 100 μL, at a controlled flow rate of 10 $\mu L \cdot min^{-1}$.

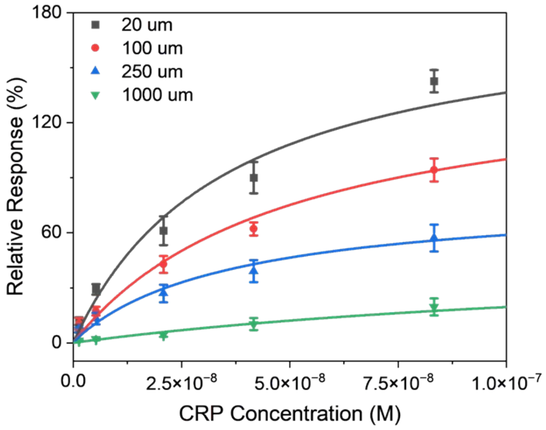

Fig. S16 Equilibrium redox capacitance (C_r in plateau) of anti-CRP/PANI electrodes as a function of CRP concentration, measured under varying microfluidic channel heights (20 μm, 100 μm, 500 μm, and 1000 μm) at a flow rate of 10 $\mu L \cdot min^{-1}$. Data points were fitted to Langmuir isotherms to derive association constants (K_a). Error bars represent the standard deviation from three individual electrodes (n = 3).

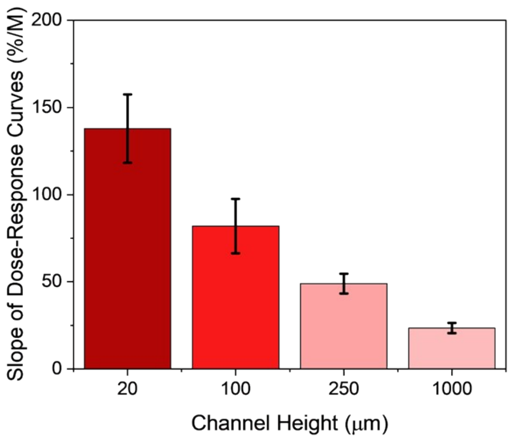

Fig. S17 Slope of "Dose-Response" curves, determined from the logarithm of CRP concentration ($\log c_{CRP}$) versus equilibrium redox capacitance (C_r in plateau), as a function of microfluidic channel heights (20 μm, 100 μm, 500 μm, and 1000 μm) for CRP and anti-CRP biorecognition. Error bars represent the standard deviation from three individual electrodes (n = 3). The results indicate enhanced sensitivity with decreasing channel height, highlighting the impact of microfluidic confinement on improving analyte recruitment efficiency.

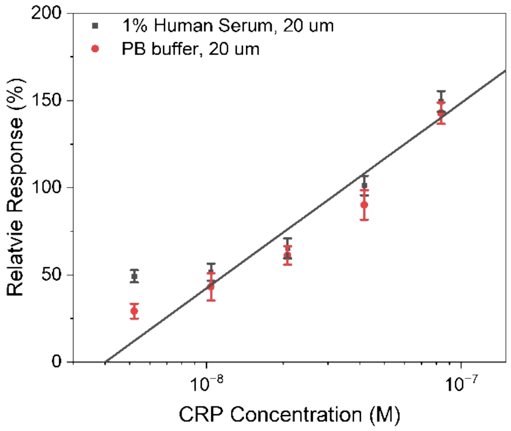

Fig. S18 "Dose-Response" curves for the assaying of CRP spiked in 1% human serum (black) and PB buffer (red) within a 20 μm microfluidic channel at an unblocked receptive PANI interface. A Limit of Detection (LoD) of 4.0 nM was noted. The recovery of CRP spiked in serum was averaged across concentrations of 1.25, 2.50, 5.00, and 10.0 $\mu g \cdot mL^{-1}$ (Fig. **6b**). Error bars represent the standard deviations from three individual electrodes (n = 3).

Reference

1. A. Baradoke, R. Hein, X. Li and J. J. Davis, Reagentless redox capacitive assaying of C-reactive protein at a polyaniline interface, *Anal. Chem.*, 2020, **92**, 3508-3511.
2. S. Kang, M. Sharafeldin, S. C. Patrick, X. Chen and J. J. Davis, Ultrafast biomarker quantification through reagentless capacitive kinetics, *Anal. Chem.*, 2023, **95**, 4721-4727.
3. M. Srisa-Art, E. C. Dyson, A. J. deMello and J. B. Edel, Monitoring of real-time streptavidin- biotin binding kinetics using droplet microfluidics, *Anal. Chem.*, 2008, **80**, 7063-7067.
4. T. M. Squires, R. J. Messinger and S. R. Manalis, Making it stick: convection, reaction and diffusion in surface-based biosensors, *Nat. Biotechnol.*, 2008, **26**, 417-426.
